# Reintroducing *Seminars in Immunopathology*: A renewed platform for translational science

**DOI:** 10.1007/s00281-026-01086-9

**Published:** 2026-09-16

**Authors:** Petra Clara Arck

**Affiliations:** 1 Division of Experimental Feto-Maternal Medicine, Department of Obstetrics and Fetal Medicine, University Medical Center Hamburg-Eppendorf, Hamburg, Germany; 2 Hamburg Center for Translational Immunology, University Medical Center Hamburg-Eppendorf, Hamburg, Germany

It is with great enthusiasm that we reintroduce *Seminars in Immunopathology* to the scientific community, a journal with a distinguished legacy that is now entering an exciting new phase of growth, inclusivity, and scientific impact. As the field of immunopathology continues to evolve at an accelerating pace, so too must the platforms that support its dissemination. Our renewed vision reflects this reality: to serve as the premier destination for high-quality, clinically relevant, and translational research that advances the understanding and treatment of immune-mediated disease.

One of the most significant changes in this new chapter is our transition from a predominantly solicited-content model to an open submission system. While commissioned contributions have historically enabled focused discussions of key topics, we recognize that scientific innovation frequently emerges from diverse perspectives and unexpected directions. By opening our doors to submissions of review articles from the broader research community, we aim to capture the full breadth of scientific discovery occurring across immunology, pathology, and related disciplines. This evolution will not only broaden participation but also strengthen the diversity, relevance, and impact of the research we publish.

At its core, *Seminars in Immunopathology* remains committed to publishing review articles that bridge fundamental immunology and clinical application. We are particularly interested in translational research that sheds light on disease mechanisms and informs new diagnostics, therapies, and approaches to patient care. From autoimmunity and chronic inflammatory disorders to cancer immunology, host-pathogen interactions, emerging immune-mediated diseases, developmental origin of immune diseases, reproductive disorders and systems immunology approaches, our goal is to showcase science that deepens biological understanding while driving meaningful clinical advances.

Maintaining the highest standards of quality is central to this mission. Every manuscript will continue to undergo rigorous peer review to ensure scientific robustness, novelty, and relevance to the field. At the same time, we are equally committed to providing an outstanding author experience. Authors deserve not only a respected venue for publication but also a fair, transparent, and efficient editorial process. To that end, we are refining our workflows to deliver competitive turnaround times, clear communication, constructive feedback, and a review process that recognizes the considerable effort invested in every submission.

A key pillar of this renewed vision is our expanded and reinvigorated Editorial Board. We have brought together a broader, internationally representative group of distinguished Medical and Clinician scientists whose expertise spans the full spectrum of modern immunopathology. This expansion strengthens our scientific coverage, enables deeper engagement with emerging areas of research. Equally important, our editors share a commitment to scientific rigor, editorial excellence, integrity, and service to the research community. Their leadership will help shape the journal’s future and ensure that it remains at the forefront of the field.

**Presenting concise details: Mini-reviews**

We are also pleased to introduce a new article format: the Mini Review. Designed to be concise, focused, and highly accessible, Mini Reviews provide expert perspectives on rapidly evolving topics, novel biological mechanisms, and emerging therapeutic opportunities. This invitation-only format will allow readers to quickly gain insight into developing areas while offering authors a flexible platform to highlight important advances and future directions. We believe Mini Reviews will become an important resource for both specialists and the wider biomedical community.

**Moving forward**

As we embark on this new chapter, our ambition is clear: to strengthen *Seminars in Immunopathology* as a leading journal in the immunopathology space, recognized for excellence in translational science, editorial rigor, author service, and research integrity. We seek not only to publish outstanding science but also to cultivate a trusted scholarly community founded on quality, transparency, innovation, and trust.

We invite researchers from around the world to join us in this endeavor by submitting reviews on topics outlined above. Together, we will continue to advance the understanding of immune-mediated disease and ensure that the discoveries shaping tomorrow’s diagnostics and therapies are communicated through a journal dedicated to scientific excellence, integrity, and lasting impact. 

